# A stochastic structured metapopulation model to assess recovery scenarios of patchily distributed endangered species: Case study for a Mojave Desert rodent

**DOI:** 10.1371/journal.pone.0237516

**Published:** 2020-08-13

**Authors:** Stephanie T. Castle, Patrick Foley, Deana L. Clifford, Janet Foley

**Affiliations:** 1 Department of Medicine and Epidemiology, School of Veterinary Medicine, University of California Davis, Davis, California, United States of America; 2 Wildlife Investigations Lab, California Department of Fish and Wildlife, Rancho Cordova, California, United States of America; 3 Department of Biological Sciences, Sacramento State University, Sacramento, California, United States of America; The University of Southern Mississippi, UNITED STATES

## Abstract

While metapopulation theory offers tractable means to understand extinction risks for patchily-distributed endangered species, real systems often feature discrepant patch quality and accessibility, complex influences of environmental stochasticity, and regional and temporal autocorrelation. Spatially structured metapopulation models are flexible and can use real data but often at the cost of generality. Particularly as resources for management of such species are often critically limited, endangered species management guided by metapopulation modeling requires incorporation of biological realism. Here we developed a flexible, stochastic spatially structured metapopulation model of the profoundly endangered Amargosa vole, a microtine rodent with an extant population of only a few hundred individuals within 1km^2^ of habitat in the Mojave Desert. Drought and water insecurity are increasing extinction risks considerably. We modelled subpopulation demographics using a Ricker-like model with migration implemented in an incidence function metapopulation model. A set of scenarios was used to assess the effect of anthropogenic stressors or management actions on expected time to extinction (T_e_) including: 1) wildland fire, 2) anthropogenically-mediated losses of hydrologic flows, 3) drought, 4) intentional expansion of existing patches into ‘megamarshes’ (i.e. via restoration/enhancement), and 5) additive impacts of multiple influences. In isolation, marshes could be sources or sinks, but spatial context within the full metapopulation including adjacency could alter relative impacts of subpopulations on all other subpopulations. The greatest reductions in persistence occurred in scenarios simulated with impacts from drought in combination with fire or anthropogenically-mediated losses of hydrologic flows. Optimal actions to improve persistence were to prevent distant and smaller marshes from acting as sinks through strategic creation of megamarshes that act as sources of voles and stepping-stones. This research reinforces that management resources expended without guidance from empirically-based modeling can actually harm species’ persistence. This metapopulation-PVA tool could easily be implemented for other patchily-distributed endangered species and allow managers to maximize scarce resources to improve the likelihood of endangered species persistence.

## Introduction

Metapopulation modeling is an important tool in conservation of endangered species in fragmented landscapes, particularly those that are dependent on climate-impacted resources such as water [1, 2]. Essentially, a metapopulation is an emergent property of inter-dependent subpopulations, each experiencing asynchronous extinction and recolonization [3, 4]. Early metapopulation models were mathematically tractable and intuitive, yet arguably inadequate to account for dynamics in most natural systems [5]. Spatially structured metapopulation models and incidence function approaches injected realism, but at a cost to the elegant symmetry of the Levin’s metapopulation in the strict sense. Real management problems feature discrepant patch quality and accessibility, complex influences of environmental stochasticity on patch quality, lack of data on predictors of population demography, and regional and temporal autocorrelation of metapopulation parameters. Theorists have tackled these problems and applied their innovations to exemplar species (e.g. stochastic dynamic programming of the Mount Lofty Ranges Southern emu-wren, *Stipiturus malachurus intermedius* [6]), showing that, beyond simplicity, metapopulation theory’s exceptional strength is its flexibility and ability make use of real data.

The critically endangered Amargosa vole (*Microtus californicus scirpensis*) is a microtine rodent occupying less than 40 hectares of rare and deteriorating marsh habitat in the Amargosa River Basin. Like numerous geographically isolated endemic species, the vole is at extreme risk of extinction by virtue of very small population size and strict dependence on patchy marsh habitat within the driest portion to the Mojave Desert [7–9]. Their small population size makes them vulnerable to local environmental pressures, environmental stochasticity, inbreeding, and demographic challenges [10–13]. Nearby growing cities and towns, agriculture, and solar energy farms compete for the critically limiting resource–water, and water is increasingly unreliable due to anthropogenic climate change and drought [14, 15]. Climate change projections suggest that, among California’s ecosystems, inland deserts are likely to experience the most severe impacts. The Mojave ecoregion is projected to experience of loss of habitat suitable for present vegetation from 83 km^2^ at present to only 9–59% of that [16, 17] Normalized difference vegetation index (NDVI) data reveal decades-long attrition of water, decreased plant production, smaller habitat area, and less connectivity among habitat patches, all exacerbated by changing climate [12, 18]. Countering such challenges requires conservation resources to support and improve habitat, optimally guided by theory and data.

Here we developed a flexible, spatially-explicit stochastic metapopulation model that uses available data and allows managers to assess interventions or unintended external influences on habitat patches and corridors. Specifically, our goals were to: 1) to develop the metapopulation viability analysis framework, 2) parameterize the model using data on patch quality and configuration, environmental impacts, vole demographic data, and inter-patch migration events, 3) classify patches in a source-sink-transition framework depending on whether each patch is expected to persist >25 yr, or if persistence is contingent on patch position within the metapopulation, and 4) analyze how changes in water availability and habitat affect extinction risk of the metapopulation; those simulated changes included status quo, projected climate change without intervention, climate change with intentional expansion of existing patches into ‘megamarshes’ (i.e. via targeted restoration/enhancement), and enforced local provision of stable water. In the face of limited resources, metapopulation modeling can efficiently prioritize strategies for conservation of species in fragmented landscapes, particularly those that are highly dependent on climate-impacted resources such as water.

## Methods

### Study system

**Fig 1:**
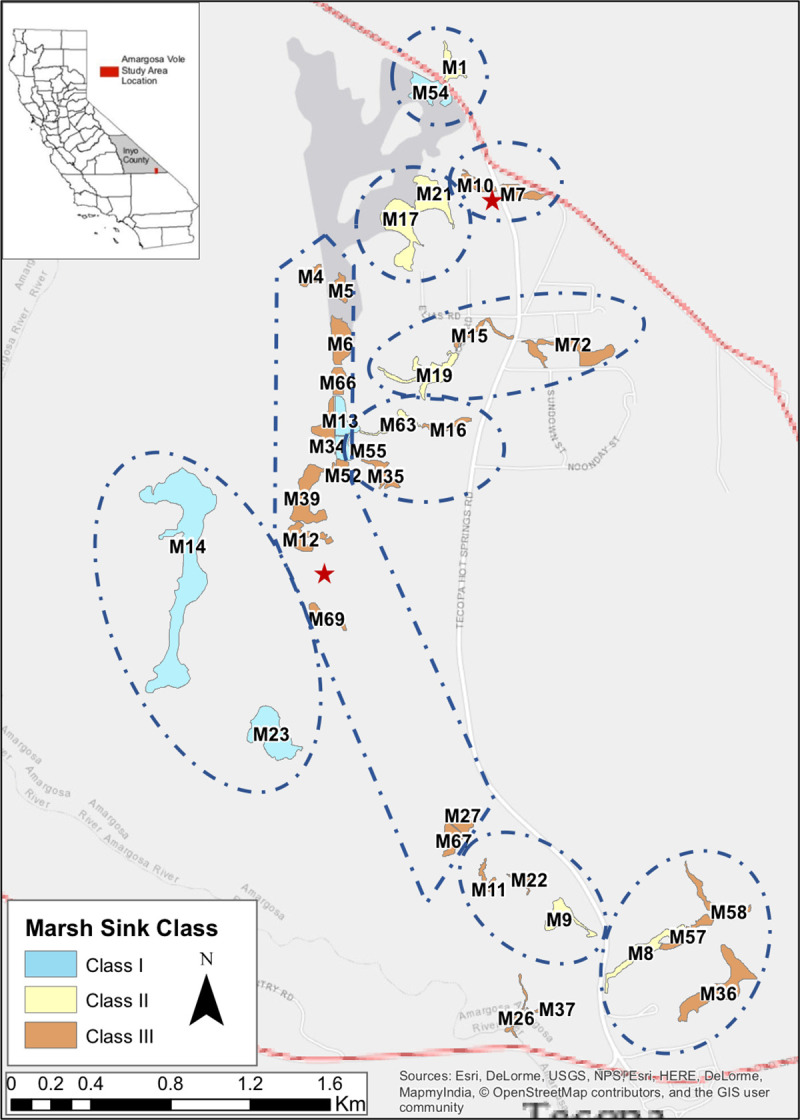
Range map of the Amargosa vole. Map of range and marsh habitat patches of the Amargosa vole, *Microtus californicus scirpensis*. Marshes are numbered according to conventions of the ad hoc Amargosa vole team. Groups of marshes are outlined based on shared dependency on original water sources. Red stars indicate placement of IS1001^TM^-12V Biomark RFID transceiver and integrated corded antennae. Sink class I has a predicted time to extinction (T_e_) > 25 yr; class II T_e_ = 10–25 yr, and class III T_e_ < 10 yr.

The Amargosa River basin, in the driest portion of the Mojave ecoregion, features numerous small relict wetlands from the Pleistocene, 10,000 years before modern water recession and habitat change. These wetlands are biodiversity hotspots with endemic or endangered springsnails (*Pyrgulopsis* sp.), pupfish (*Cyprinodon* spp.), Amargosa niterwort (*Nitrophila mohavensis)*, Tecopa birdsbeak (*Corylanthus tecopensis*), Bell’s least vireo (*Vireo bellii pusillus)*, and the Amargosa vole. The total range of Amargosa voles includes 36 marshes across less than 1km^2^ of patchily distributed habitat (Fig 1) near the lower Amargosa River from 35.8492 to 35.8863 latitude and -116.2170 to -116.2477 longitude [10, 19]. Average daytime temperatures range from 15–23°C in winter and 37–43°C in summer. Mean annual precipitation (1972–2011) is 120 mm [20]. Fewer than 1000 Amargosa voles remain in the wild [12].

The wetlands depend on surface and subterranean water from precipitation on nearby mountains (averaging 700 mm/year at high elevations [21]) and patchily distributed springs tapping a fossil aquifer, an inadvertently created Artesian well, and overflow from wells and graywater from nearby homes and bath-houses [22]. Few of the marshes depend solely and directly on natural water flows, but rather are highly dependent on human water use. Typically, one marsh occurs at a spring or pump outflow source, and the marsh’s outflow serves downstream marshes which we grouped by water sources [12] (Fig 1). The vole relies on bulrush (*Schoenoplectus americanus*)-dominated habitat for food as well as refuge from predators and thermal extremes [13, 19, 23]; such marshes occur as patches within a harsh alkaline desert matrix.

### Field methods

Mean water depth of each marsh patch was calculated from water depth measurements collected at grid-point locations every 20m throughout bulrush-dominated portions of each marsh. Water depth measurements were collected twice (peak winter and summer) during 2017 to capture seasonal changes in water levels although overall water levels could change annually as well, particularly under impacts of drought.

Voles were trapped in a nested grid design for population size estimates [13], and later along comparably established grids to detect movement. Records of tagged vole movements between marshes were compiled from live trapping during this study and from studies in 2013–2017 [12, 13, 24–27]. Movement was also monitored using RFID transceivers and integrated corded antennae (IS1001^TM^-12V, Biomark; S1 File). Transceiver units were strategically placed between paired marshes 7/10 and 69/12, separated by a clear pinch point (Fig 1). Live-trapping occurred in paired marshes described above every 6–8 weeks during from February 2017- August 2018, and each individual captured was marked with a uniquely numbered metal ear tag (National Band and Tag, Newport, KY) and received a 12 mm passive integrated transponder (PIT) tag (Biomark, Boise, ID). Inter-marsh movement was recorded when a PIT-tagged animal passed within 36 cm of antennae; these antennae were positioned so as to allow us to infer animals leaving or entering a marsh. Transceivers recorded movement of tagged voles from June 2017 –August 2018. All live trapping of animals was performed pursuant to California Department of Fish and Wildlife Scientific Collecting Permit #SC854, US Fish and Wildlife Service Amargosa vole Recovery Permit #TE546414A-2, UC Davis Institutional Animal Care and Use Committee Protocol #19905, and an MOU with the Bureau of Land Management.

### Model overview

The main model module, *metavole*.*R*, was constructed in R using a spatially structured metapopulation model approach related to the incidence function approach [28, 29]. The stochastic model was implemented as a Monte Carlo simulation with 1-year time steps. The metapopulation landscape (*volescape*) was a dataframe representing 36 circular marshes with area and centroid characteristic of natural vole habitat. As any metapopulation reflects a balance between local extinctions and colonization, we estimated both of these.

Local extinctions occurred when N_i_ < 1; predictions of N_i_ were derived from Ricker dynamics, i.e. $N_{t+1}=N_{t}\times {exp}^{\left(rd(1-N_{t}/K)+\varepsilon \right)}$, where N_t_ was patch subpopulation size at time = *t*, *r*_*d*_ was per-capita subpopulation growth rate at low population density, K_*i*_ was subpopulation carrying capacity, and ε was environmental stochasticity (a normal random variable with mean = 0 and variance = *v*_*r*_ [30]).

Colonization was simulated to be a function of inter-patch distance and the probability that voles would emigrate from any given patch. Each patch *i* was subject to a particular “migration pressure” function [28] which was a Poisson random variable. Poisson random variables are characterized by a single parameter (mean and variance) λ; λ was defined for the metapopulation as *β*∑(*N*_*j*_×*e*^−*αd*^), i.e. it is a compositive function comprising subpopulation sizes of all marshes *j* ≠ *i*, the inverse of the mean dispersal distance (α), inter-patch distances (d) from the center of each marsh *j* to the center of marsh *i*, and a calibrator (*β*) [4]. There is little discussion of the parameter β in the literature, and we are not aware of it having received a name previously. We describe our approach to choosing its value, how sensitive model output is to the value we chose, and how this impacts model applicability and future research in subsequent sections of the paper.

The simulation was run for a given number of years (a trial), during which vole demography in each patch was simultaneously simulated according the Ricker dynamics, which yielded whether or not that patch was extinct and if not extinct, its N_i_. The subpopulation N_i_ values then were used to calculate migration pressures for each patch, which represents the function that allowed for inter-patch migration. Once the algorithm had repeated these events the desired number of trials, the simulation performed accounting to create a history for all patches at each year of: mean and variance of N, total metapopulation size, the number of migrants, the expected number of years before the metapopulation would go extinct (time to extinction, T_e_), and each patch’s occupancy (S1 Table). All data and software code are available from the authors upon request.

### Model parameterization

Parameter definitions, data sources, and values are summarized in Table 1. Bayesian prior estimates of population growth (*r*_*d*_) and variance of environmental stochasticity (*v*_*r*_) from better-studied California vole subspecies were used; these were calculated from linear regressions of time series of population size data [9], available at https://foleylab.vetmed.ucdavis.edu/resources.

**Table 1:** Parameter values in *metavole*.*R*. Objects used in *metavole*.*R*, a spatially structured metapopulation model of Amargosa voles. N/A indicates a data source is not applicable for a given object.

Object	Default (units)	Description	Source
*metavole*.*R*			
N_i_	initialized at 1×K_volume_×area	Subpopulation size at marsh *i*	This study
*r*_*d*_	0.01/(vole×yr)	Instantaneous growth rate of the population	[9]
K_volume_	17 voles/(cm×ha)	Proxy for per-patch carrying capacity and quality–calculated as published marsh population estimate/(mean marsh water depth × area)	This study
*v*_*r*_	1 (vole×yr)^2^	Variance of environmental stochasticity	[9]
spacecor	0	Degree to which *r*_*d*_ and K co-vary between adjacent marshes, from 0 (no correlation) to 1 (completely correlated)	N/A
*β*	0.01	Migration calibrator	[28]
*Α*	0.01/m	Inverse of the mean dispersal distance among marshes	This study
d_ij_	M	Distance between centers of patches i and j	Values determined from ArcGIS
volescape	Volescapepf	The landscape of 36 marshes characterized by marsh number, area, water volume, and geographical centroid. Alternate versions of volescapepf used for scenarios as described in S2 Table.	This study
*metashell*.*R*			
tmax	101	Number of years the simulation runs	N/A
trials	500	Number of times *metavole*.*R* simulation is run	N/A

The model module *metavole*.*R* required values of quality for each patch and the Ricker model for within-patch population dynamics required estimates of population growth and carrying capacity. In foundational incidence function metapopulation literature, patch quality was considered to be best predicted typically by area [28]. However, we aimed to assess multiple different predictors of patch quality (area, water depth, area × water depth = volume), and did so by choosing the linear regression model with the highest R^2^ among candidate predictors of patch vole population size (N_i_). We assumed that most of the N_i_ were at or near their carrying capacity (K_i_), because voles in occupied marshes would be expected to have high fecundity and ability to rapidly achieve maximum population size but are limited by patch area and connectivity.

Migration pressure was estimated by using a plausible value for β (while it is typically set to 1 for locally common species or at least those that can access patches readily [28], we chose a much lower value because neither characteristic applies to Amargosa voles), α calculated from observed inter-marsh movement events recorded in this study (see ‘Field methods’), and distance between patches (d_ij_) determined from maps. A plot of cumulative number of voles traveling each distance indicated exponential decay (S1 Fig) with rate (the maximum likelihood estimator for α) = 1/(mean known distance travelled).

### Trapping and demographic analyses

Results of trapping and recapture analysis to determine population density (D) of voles have been published previously [13, 31]. Trapping was undertaken over six months in 2012 and 2015 at six randomly-placed, consistently-configured grids of 108 traps/grid. Each grid was 105 m x 95 m in dimension with twelve 15 m x 15 m subgrids nested within the grid and each subgrid consisted of nine trap stations in a 3 x 3 arrangement with the traps spaced 7.5 m apart. The grids spanned available habitat types and were trapped for four consecutive days and then resampled monthly. In the original reports [13, 31], data were analyzed with several different models: the boundary strip [32, 33] conditional likelihood model with two parameters (probability of first capture (p) and probability of recapture (c)) and the spatially-explicit capture-recapture method (SECR) [34–36]. Boundary strip models were compared using the bias corrected version of Akaike’s Information Criterion (AICc) [37] allowing both parameters to vary over time, only one to vary but the other not to vary, and allowing neither to vary. SECR models were also compared with AICc, including: (1) no effect on the detection function g(d) (probability of capture at a given distance from the center of the home range) due to temporal, behavioral, or individual variation; (2) g(d) influenced by temporal variation but not behavioral or individual variation; (3) g(d) influenced by behavioral variation but not temporal or individual variation; and, (4) g(d) influenced by individual variation but not temporal or behavioral variation. The package RMark [38] was used to estimate D for boundary strip models and the package secr [39] was used for the SECR models.

Population size was calculated from D and total marsh area. All models gave similar estimates of population size, with the best supported model being the SECR model with no variation in g(d). Subpopulation sizes at 14 well-studied marshes were then estimated from quarterly, range-wide trapping done between Nov 2015-Sept 2016 [40]. Density was estimated using SECR as done for the initial six grids.

Finally, we created a general rule for optimal prediction of N_i_ and K_i_ using multiple linear regression with area, depth, and volume predictors as described above. The optimal model was chosen by stepwise removal of predictors to minimize AIC. Because the optimal model retained only volume, we therefore calculated K for each marsh as K_i_ = (water depth × marsh area × K_volume_), where K_volume_ = N_i_/(water depth (cm) × marsh area (ha)) and is an analogue to subpopulation density at K.

### Sensitivity analysis

Sensitivity analysis was conducted using the One Factor at a Time technique [41], exploring T_e_ across a plausible spectrum of input values. The model was run for 500 trials, each of 101 years. Variables assessed were: a) *r*_*d*_ from -0.5 to 0.5 in 0.1 increments, b) K_volume_ from 1 to 100 in increments of 5, c) *v*_*r*_ from 0 to 10 in increments of 1, d) *β* from 0 to 0.1 in 0.01 increments, and e) α from 0 to 0.1 in 0.01 increments.

### Modeling scenarios

Habitat patches have been classified as source if *r*_*d*_ > 0 and sink if *r*_*d*_ <0 [42]; here we updated definitions to: 1) depend explicitly on expected subpopulation persistence over 25 yr, 2) allow for each subpopulation’s growth rate to range from positive to negative due to stochasticity if close to 0, 3) contextualize a patch as source or sink based on its proximity and accessibility to other patches, and 4) add an intermediate class of inconsistent sink or source patches. The patch types are: 1) Context-independent Source where *r*_*d*_ > 0 and T_e_ tended to be high with the subpopulation persisting in *metavole*.*R* even when isolated (i.e. no migration from other patches), 2) Rescued Sink, which typically had *r*_*d*_ <0 and low T_e_ in isolation but which, likely due to a form of rescue effect (here via colonization) [43], frequently maintained a resident subpopulation when in the metapopulation, 3) Context-independent Sink with *r*_*d*_ <0 and low T_e_ both in isolation and in the metapopulation, and 4) Converted Sink, where a patch that had functioned as a source became a sink in the context of the metapopulation. Each of the 36 marshes was classified by running the simulation setting all marshes except the query marsh to an area × water depth of 0 and then evaluating T_e_ for that marsh in isolation.

Scenarios were run to assess ecologically relevant anthropogenic stressors and management actions including: 1) wildland fire, 2) anthropogenically-mediated losses of hydrologic flows, 3) drought (minor, moderate, and severe), 4) strategically located ‘megamarshes’ in the northern, central, or southern habitat range (indicative of potential habitat restoration/enhancement projects), and 5) additive drought with fire, anthropogenic water loss, and megamarsh creation. Water reduction scenarios would directly reduce or eliminate habitat for this marsh-dependent species. Impacts of fire are less clear, especially over time, but immediate impacts would be to remove burned habitat from potential occupancy, as fire would remove food, canopy protection against aerial predators, and litter sites where voles escape predators and locate their nests. Each scenario was implemented by adjusting patch area and water volume to increase or decrease patch-specific carrying capacity (K_*i*_) (S2 Table). Default model parameters remained static except where noted to implement the scenario; each scenario represented a model run of 101 years and 500 trials of the single set of input conditions that defined that scenario. Model outputs included a history of the subpopulation sizes at each marsh updated annually, mean subpopulation size across all marshes, mean metapopulation size (total number of voles), number of occupied patches, number of migrants, and the estimated time to extinction (T_e_) after *t* years.

### Statistical analysis

Data were maintained in Excel (Microsoft, Redmond, WA) and ArcGIS (ESRI, Redlands, CA), and analyzed in R [44]. We evaluated whether sex was associated with mean distance traveled using a Student’s t-test and whether season, corridor vegetation status (vegetated, non-vegetated, or mixed), and type of corridor vegetation (bulrush, salt grass, and predominantly or partially bare) were associated with distance using ANOVA. To evaluate management scenarios, we compared means of T_e_ (mvte), proportion of occupied patches at *t* = 25 years (mvp25), total population size at *t* = 25 years (mN25), and the number of migrants across patches at *t* = 25 years (mcol25) using one-way ANOVA and Tukey HSD mean separation. Linear regression was used to explore relationships between response variables and increasing levels of drought. Values were considered significant if p ≤0.05.

## Results

### Parameter estimates

Values for *r*_*d*_ and *v*_*r*_ were calculated from other California vole subspecies as described by Foley and Foley (9), yielding averages for each of 0.01 and 1.0, respectively (Table 1). Such a Bayesian approach was used because our time series calculations require either a Bayesian prior or multiple successive years of data which have not yet been acquired for the Amargosa vole. The value for *r*_*d*_ we used compares with monthly values of *λ* for Amargosa voles ranging from a low of 0.5 in November of 2014 and a high of 2.3 in June 2014, which correspond to *r*_*d*_ = *ln*(*λ*) = -0.69 to 0.83. The coefficient from the best-fitting regression model of N_*i*_ (values given in Table 2) on water depth x patch area was 33.39, providing a calculation of K_volume_ as 17 voles/(cm x ha) (Table 2). We used a default value of *β* = 0.01 for all simulations except where otherwise stated (e.g. in sensitivity analysis). The observed mean distance voles moved among marshes was 136.86 m and the MLE for decay rate of distance travelled was 0.0073. For modeling, this number was rounded to α = 0.01. Summary statistics were compiled for the movement data (Table 3). Sex was not significantly associated with movement distance, although a single female was an outlier with a distance moved of 478 m. However, once she was removed from the dataset, mean distances moved by males and females were almost exactly the same (47 m); we calculated α with this outlier removed. All other independent variables assessed were significant, with increased distance of movements during winter and spring, along corridors that were not vegetated, and when crossing salt grass or open playa.

**Table 2:** Amargosa vole subpopulation sizes (N_i_) at 14 well-studied marshes and best-fitting regression model of N_*i*_ on water depth x patch area.

Marsh number	N_i_		
1	120		
2	23		
3	7		
4	1		
6	8		
10	20		
12	7		
12	1		
21	18		
23	19		
39	83		
54	113		
67	10		
Model	R^2^	**AIC**	**p-value**
N = 0 + ß_3_ area + ß_2_ volume + *ε*	0.847	78.61	N/A
N = 0 + ß_1_ volume + *ε*	0.834	77.77	2.0x10^-6^

**Table 3:** Summary of movement patterns of 22 Amargosa voles that moved between different marshes near Tecopa, California.

Factor	Frequency (No. of movements)	Mean distance moved (m)		Significance (*t* or *F*, df, p)
Sex				*t* = 0.9, df = 9.2, p = 0.37
Male	141	47.4	±59.2	
Female	10	90.5	±1436.0	
Female ^a^	9	47.4	±48.5	
Season				*F* = 3.7, df = 3, p = 0.01
Winter	15	74.9	±104.4	
Spring	18	90.3	±85.2	
Summer	97	37.5	±42.7	
Fall	21	65.7	±100.5	
Vegetated corridor				*F* = 107.3, df = 2, p < 0.001
No	3	321.0	±140.5	
Yes	145	39.2	±39.7	
Mixed	4	257.3	±75.9	
Type of vegetation				*F* = 133.9, df = 2, p < 0.001
Bulrush	144	37.7	±36.0	
Salt grass	6	240.5	±0.7	
Bare or bare/mixed	2	297.8	±110.9	

^a^ Mean distance moved by females with outlier removed.

### Sensitivity analysis

**Fig 2:**
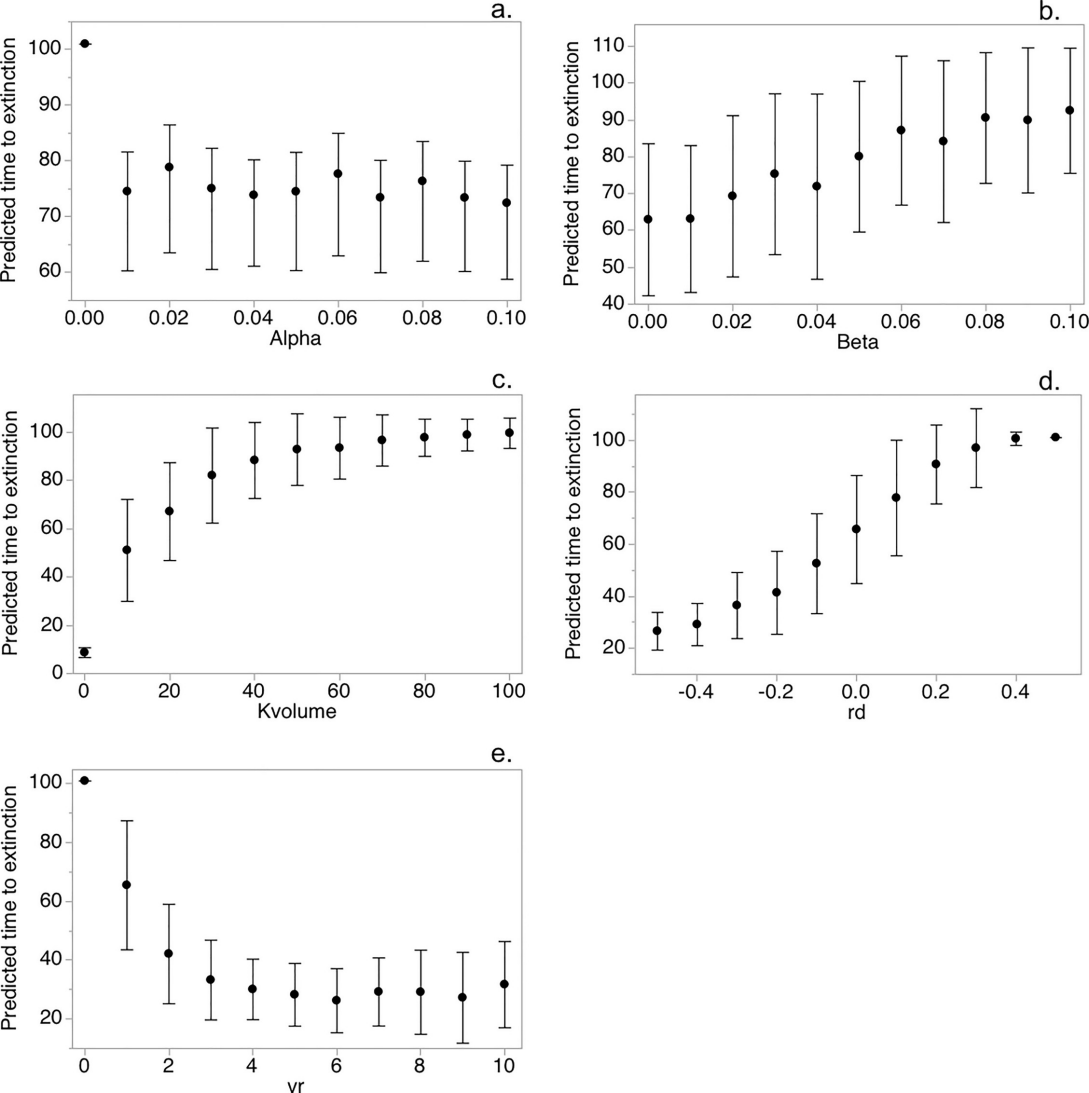
Sensitivity analysis plots. Sensitivity of predicted times before Amargosa voles are extinct (T_e_) on a spectrum of values for five parameters in a simulation model *metavole*.*R*. Parameters including (A) mean dispersal distance, *α*, (B) migration calibrator, *β*, (C) patch carrying capacity, K_volume_, (D) estimated rate of population growth, *r*_*d*_, and (E) environmental stochasticity, *v*_*r*_, are described in the text. Graph gives mean Te and standard deviation.

Spectra of plausible values of the parameters in *metavole*.*R* were explored, yielding predictable changes in mean T_e_ across trials as a function of each input. T_e_ was an approximately linear function of *r*_*d*_ and *β*, though there was evidence in the former of a weak sigmoidal curve tending to reach a plateau near *r*_*d*_ = 0.4 (Fig 2D). Exponential decay was apparent in the relationships between T_e_ versus *v*_*r*_ and *α*. In both cases, any increases in the independent variables would be predicted to result in sharp reductions in metapopulation persistence. Lastly, increases in K_volume_ improved T_e_ linearly until eventually saturating at approximately K_volume_ = 40, although this was due to the limited run of the simulation at 101 years.

### Per-patch impact on metapopulation

**Fig 3:**
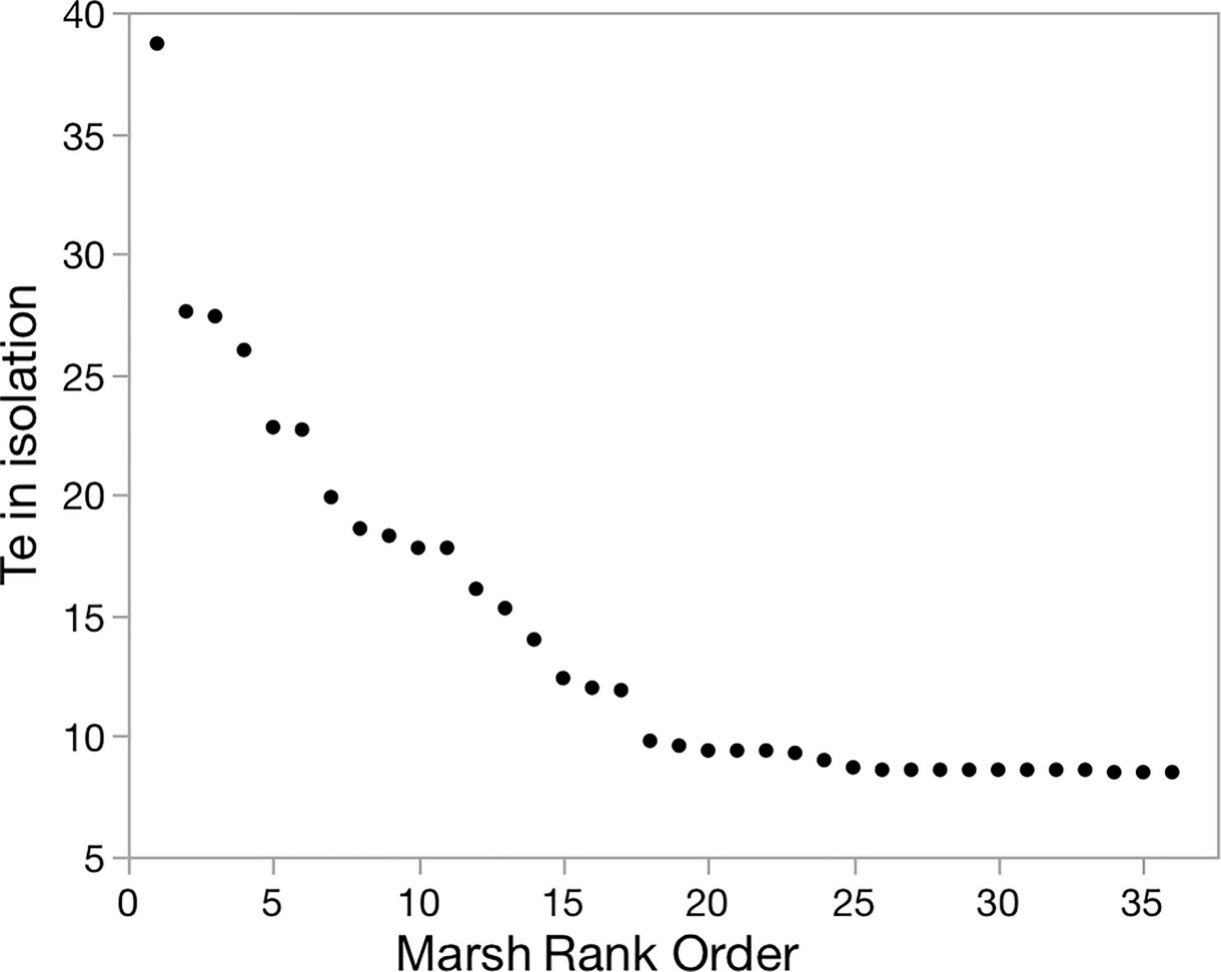
Estimated time to extinction for individual patches. Occupied marshes near Tecopa, California ranked by predicted times to extinction when subpopulation dynamics were simulated using the model *metavole*.*R*, assuming no other voles from outside marshes could access the query marsh (marsh in isolation).

Subpopulation dynamics were simulated for each marsh in isolation, setting values of all other marshes to 0, yielding a distribution of T_e_ values for individual marshes ranked by decreasing values of T_e_ (Fig 3). Four marshes were Context-independent Sources with relatively high T_e_ in isolation >25 yr (Marshes 54 in the north, 13 in the north-central cluster, and 14 and 23 towards the southwest in an isolated playa, Fig 1). Thirteen marshes in isolation would have T_e_ from 10–25 years and 19 marshes if isolated would persist <10 years, i.e. acting as Context-independent Sinks, many of them in the south. We then examined the behaviors of each of the marshes when the simulation was run as an intact metapopulation. Within the metapopulation, five marshes tended to have high probability of persisting > 25 yr (Marshes 13, 14, 23, 54, and 55), one of which (55) had such high persistence in part by virtue of rescue, given the relatively low T_e_ in isolation, i.e this marsh functions as a Rescued Sink. Marsh 34 also functioned as a rescued Sink. In contrast, Marshes 6 and 11 dropped from Sources to Converted Sinks.

### Anthropogenic impacts scenarios

**Fig 4:**
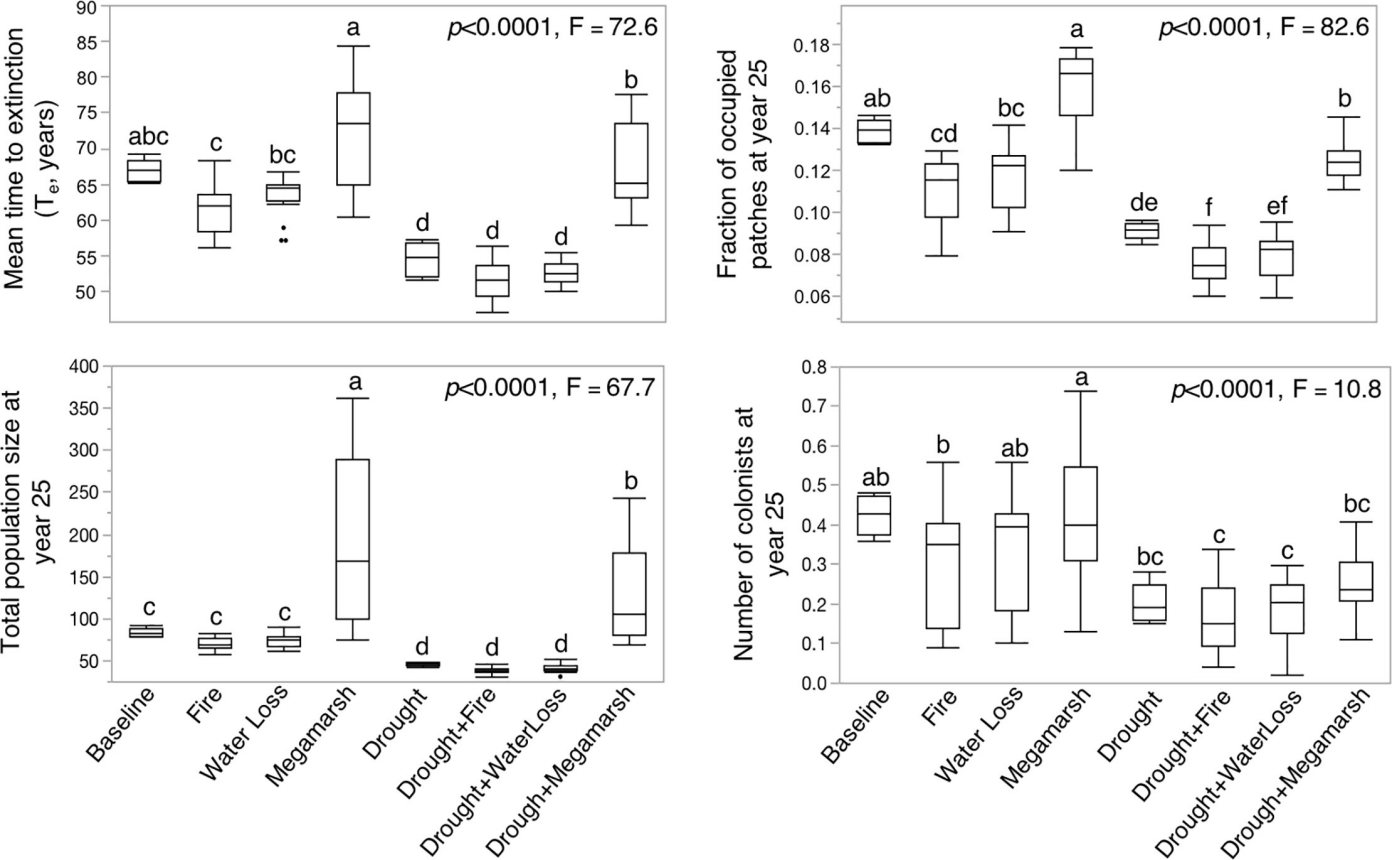
Metapopulation response to management scenarios. Mean values (and standard deviations) of metapopulation response variables across classes of scenarios simulated in *metavole*.*R* to predict impacts of habitat stressors or intervention on extinction risk. Values for F-statistic and *p*-value of ANOVA are included. Letters indicate significant differences between means for scenario classes.

**Fig 5:**
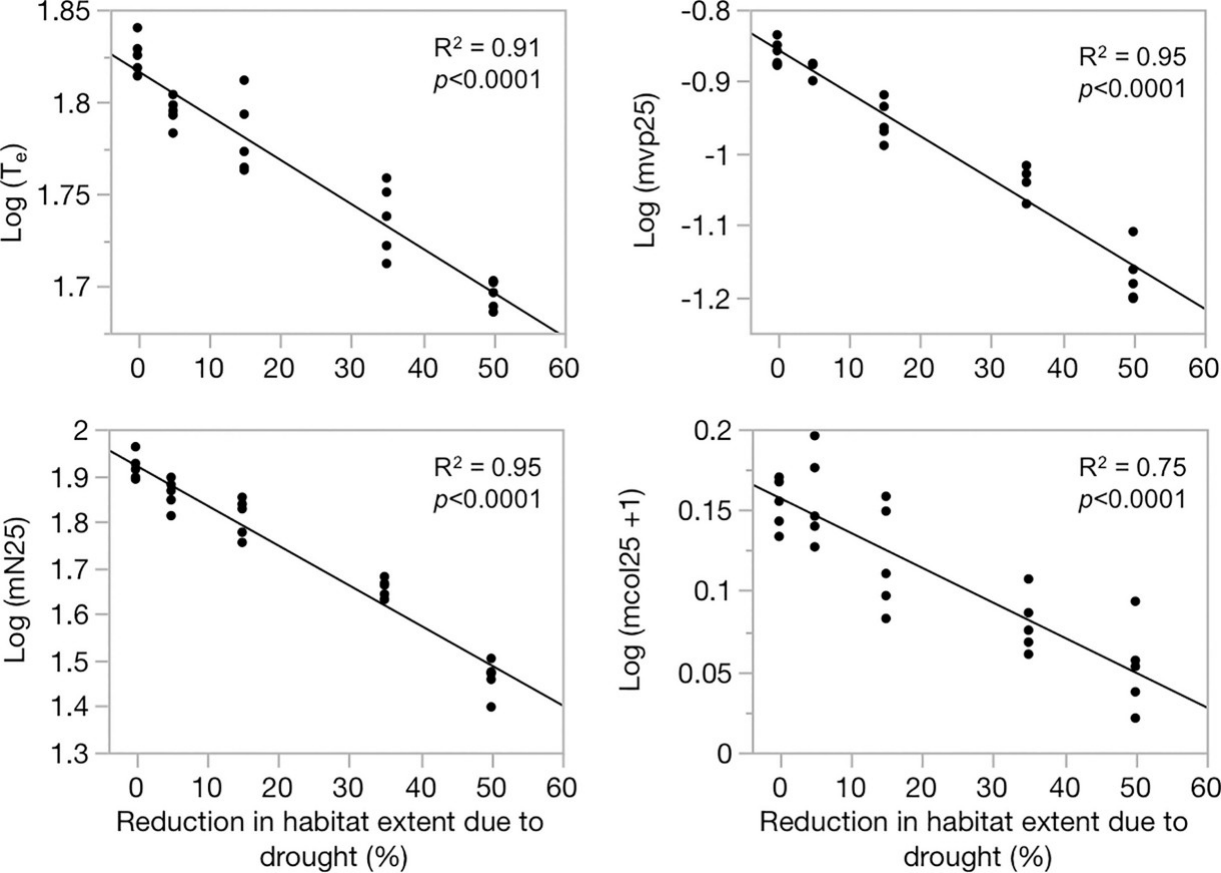
Impact of simulated drought on metapopulation dynamics. Regressions of mean expected time to extinction (mvte), mean fraction of occupied patches at time *t* = 25 years (mvp25), mean total metapopulation size at *t* = 25 (mN25), and mean number of migrants at *t* = 25 (mcol25) on percent reduction in habitat due to drought, where habitat is patch area × water depth as described in text.

Anthropogenic impacts to vole habitat were compared using *metavole*.*R*, which showed that lack of water or destruction of habitat by fire would reduce T_e_ and carefully placed megamarshes could improve T_e_, but combined effects of simultaneous drought and fire for example could be catastrophic (Fig 4, S3 Table). The T_e_ for the baseline scenario (status quo) was 67 years; addition of wildland fire (in northern, central or southern habitat extents) reduced T_e_ by 8.6% on average, anthropogenically-mediated loss of water flows reduced T_e_ by 5.4%, and moderate (35% reduction) drought reduced T_e_ by 18.4%. These differences among scenarios were significant (*p* <0.0001). The T_e_ declines significantly with increasing impact of drought (r2 = 0.91, *p*<0.0001, Fig 5). Similar trends were observed for total population size at time *t* across scenarios. Furthermore, additive impacts of Drought+Water loss and Drought+Fire were most severe, reducing T_e_ by 21.2% and 22.7% respectively, although the differences between these two scenarios were not statistically significant (Fig 4).

Trends of metapopulation sizes and migrants after 25 and 101 years mirrored findings for T_e_ across scenarios (Fig 4, S3 Table). Because the metapopulation was often extinct at 101 years during model simulations, the mean population (mNt) and mean number of migrants (mcolt) at time *t = 101* were very low (8.1 and 0.0095 respectively). For this reason, we focused on the mean total population size and number of migrants at time *t* = 25 (mN25 and mcol25). The highest number of migrants at *t =* 25 (mcol25) occurred in the baseline (status quo) scenario, and greatest reductions in mcol25 occurred in Drought+Water loss (57%) and Drought+Fire (61%) (Fig 4). The fraction of marshes occupied within the landscape at *t = 25* (mvp25) also had the greatest reduction for Drought+Fire and Drought+Water loss scenarios.

Creation of expanded ‘megamarshes’ in the landscape generally led to increases in T_e_, with northern megamarshes being particularly beneficial, increasing T_e_ 22.3% (81.8 ± 2.9 years), mvp25 by 24.0% and mN25 by 313% (Fig 4). Changes in the southern habitat region were more complex. If the entire southern region was destroyed by fire, there was no decline in T_e_ or mN25 compared to baseline conditions; however the creation of a megamarsh in the southern region led to significant increases in both T_e_ (9.5%) and mN25 (98%).

## Discussion

Amargosa voles exemplify several challenges in endangered species management, including inherent high (and difficult to predict) demographic variability, habitat specialization, dependence on fragmented habitat patches within a metapopulation, and accelerating human impacts on habitat. We have published previously a population viability analysis (PVA) that predicted persistence times for the species based on analysis of population time series [9]; here we add considerable realism and value to the PVA by explicitly incorporating landscape features, local subpopulation extinction, and inter-patch migration dynamics. Our analysis documents that no single patch in isolation could support a robust and persistent Amargosa vole subpopulation, but that the metapopulation structure itself crucially determines the source and sink qualities of each patch. The metapopulation-PVA tool is flexible, permits evaluation of possible changes to the environmental determinants of vole subpopulation success, and could easily be parameterized and implemented for other patchily-distributed endangered species. Querying the model with a set of plausible scenarios of ongoing anthropogenic disturbances or potential management interventions allows us to make recommendations that would maximize efficient use of scarce resources to improve the likelihood of Amargosa vole persistence, as is urgently advocated for other conservation programs [6, 45].

Our estimation of parameter values followed the practical guidance originally provided in Hanski’s incidence function metapopulation work [28] coupled with simple subpopulation regulation using a Ricker model and statistical assessment of best predictors for inter-patch movement and patch quality. We acknowledge that parameter values used in the present model were derived from early field research, and that ongoing research to monitor water depth and other aspects of the environment and vole status would help keep the model optimally relevant. Typically, patch area is the most intuitive predictor of patch carrying capacity in a metapopulation model, whereas we chose to use area × water depth (the optimal model based on AIC) because incorporating the biologically meaningful water depth variable better explains the difference between high- and low-quality marshes and allows us to study a key weakness and manageable characteristic of the system. However, there was little difference in AIC between a model using area and volume and one using only volume, so we chose the more parsimonious predictor (volume only).

Our model produced numerous useful output data including estimates of migrants, occupancy, and T_e_, the latter probably serving most intuitively to allow for comparison among scenarios for metapopulation responses to stressors or intervention. T_e_ has the benefit of being a single, management-relevant number for ease of statistical comparison. It resembles the “mean lifetime of a metapopulation” statistic previously proposed for stochastic and spatially heterogeneous metapopulations [46]. Sensitivity analysis supported our assumptions that changes in *r*_*d*_ and K_volume_ would have considerable impacts on T_e_, as was evident in our scenarios as well. In general, optimal provision of water remains crucial to support robust vole subpopulations and is among the most important and realistic management actions.

The sensitivity analysis also suggested that even small increases in *v*_*r*_ could have dire consequences for persistence of the Amargosa vole. There may be little managers can do to minimize *v*_*r*_, though the creation of megamarshes may buffer against stochasticity that might be more problematic in smaller patches. However, our data and modeling approach show clearly that context matters: no patches were so large and contained enough standing water as to yield independent subpopulations persisting more than 101 years. Furthermore, the relative spatial relationships among context-dependent sources and sinks critically impacted both the likelihood that an entire region would function as a sink or that a regional megamarsh would have a positive impact on overall metapopulation persistence. This dynamic is best illustrated in the southern marshes: simulated northern or central-habitat fires reduce T_e_ but we do not see that in the south, because southern fire mitigates the effects of so many sink marshes. A megamarsh in the area would improve metapopulation persistence because the context in which southern marshes are sinks is changed.

Metapopulations persist due to balanced patch persistence and accessibility. The migration parameters α and β had predictable impacts on overall T_e_ and analysis showed that impairing access to marshes (which can happen for example due to roads or loss of “stepping-stone” patches) could dramatically reduce persistence expectations. The parameter β is somewhat enigmatic and its relationship with T_e_ was linear. If a species is locally abundant and sufficiently vagile to frequently colonize new patches, it would have a high β [28] whereas neither characteristic describes the Amargosa vole at present, which is why we chose a low β value. Improving connectivity and creation of megamarshes could raise the value of β, while we also acknowledge that future studies could be done to improve the precision of our estimate of β.

In addition, intentionally reducing access to sink habitat could sometimes be a worthwhile management action if it helps prevent dead-end migrants from ending up in sinks. The full impact of such sinks depends to some extent on movement rates among marshes, which is a difficult to obtain datum, particularly for long-distance, rare, and emigrating movements, for any species [47]. We could not comprehensively catalog movement rates, but, even though earlier work suggested that Amargosa voles move minimal distances from any given capture site [13], we focused on inter-marsh movement and showed that: 1) voles will often move among patches that are adjacent if there is a vegetated or protected corridor, and 2) longer distance inter-marsh movements are rare but when they do occur, open playa does not impede movement. Our finding of longer movements along playa and salt grass is likely an artifact of this habitat patch structure because any inter-marsh habitat lacks bulrush and any movement between marsh patches require crossing playa. While our data surely failed to capture all inter-patch movement, the good fit of data to the decay curve suggests that inclusion of any additional movement events would not likely change the relationship we documented. In addition, our movement predictions correspond well with recent genetic estimates that suggested one effective colonization event per marsh per generation [11].

Migration among patches as implemented in *metavole*.*R* was determined both by inter-marsh distance and by density-dependent subpopulation dynamics, such that densely inhabited patches would be more likely to provide propagules. While one option would be to rerun within-patch dynamics whenever an animal emigrates, we did not do this in the current model because high source-patch density was a trigger for emigration. We also allowed for migration but did not assume that each migration was synonymous with a colonization. The present version did not incorporate synchronized forcers such as pulse dynamics or extreme weather events [48], although the model has the ability to address regional and temporal non-independence, an important consideration for Amargosa voles that at present persist entirely within one water catchment. An earlier study inferred that Amargosa voles persist in a metapopulation due to the habitat patchiness [10]: we were concerned that such a designation might imply that subpopulations in small patches are buffered against local extinction by colonization from nearby habitat patches. Rather, some patches may function as sinks and overall connectivity among patches may be decreasing due to reduction of local water [15]. We implemented this by classifying each patch as a Context-independent Source, Rescued Source, Context-independent Sink, or Converted Sink, extending the classifications used earlier [42].

With this innovation, scenario data must be interpreted carefully. A large proportion of marshes in the landscape is predicted to be occupied for less than 10% of the time during the 101 year time period of any simulation run and yet, even if a marsh has low occupancy, it may still provide refuge and a temporary stopping point for voles moving between patches. In the landscape of the Amargosa vole, there is a natural zone of separation between habitat patches in the northern, central and southern parts of the range. Simulated northern megamarshes have a large beneficial impact on vole persistence because they take advantage of a subset of patches that already have moderate or high occupancy rates and moderate connectivity. The megamarsh is better than the present set of nearby interconnected patches because it reduces impacts of demographic and environmental stochasticity. The central area megamarsh is less clearly beneficial, because those marshes already present in this central area are very well-connected. Only if a buffered megamarsh is installed in the south do these southern marshes do anything except inhibit overall metapopulation persistence. Thus, a very reasonable management question becomes: is it better to use scarce resources for the sure benefit derived from the northern megamarsh or to the less sure but potentially more impactful south, if both are not possible?

During a retreat in 2017, an ad hoc Amargosa Vole Team (comprising agency managers and academic and non-profit partners) listed improving water consistency, increasing habitat extent, and improving habitat quality as highest priorities for recovery of the vole. The federal Endangered Species Act and California state legislation support legal protections of water and intervention such as landscape modifications (e.g. creation of one or a few core “megamarshes” or changes to inter-marsh connectivity). Our data confirm that vegetating inter-marsh corridors would have far less benefit than improving quality of patches per se, and that megamarsh construction should only be done where it does not diminish current contributions of extant marshes but maximizes functionality of a set of adjacent marshes in a regional network or source.

Less immediately under the control of endangered species managers is water in a broad and regional sense, and yet our model shows catastrophic impacts to this species with progressively larger drought impacts. As currently implemented, exploration of water loss and fire scenarios only incorporated reduced habitat area, although we acknowledge that dispersal could also change and should be monitored. Drought combined with other anthropogenic and climate-related influences, such as fire and loss of point-source spring outflows, could have synergistic impacts on persistence of the Amargosa vole metapopulation. Even worse, drought exerts impacts on each marsh that are temporally and spatially correlated, so that our prediction of linear impacts of drought on extinction risk must actually be adjusted to allow for acceleration of those impacts. In a metapopulation analysis of highly vulnerable cloud forest in Mexico, a similar finding of regional autocorrelation of climate impacts indicated that the expectation for multiple species’ persistence was dire [49]. Ongoing loss of water in the Mojave would likely be catastrophic not just to voles but to numerous flora and fauna throughout the region [15].

Earlier research developed metapopulations of real or complex biological systems where patches are not all one size, migration is not all equal, and parameters are subject to stochasticity or nonstationarity, e.g. [6, 29, 50–53]. Some have had to omit stochasticity, assume equal patch size or accessibility, highlight that results are entirely dependent on parameter sets, or make other concessions that improve usability but reduce generality. Our incorporation of analytical Ricker dynamics within the overall simulation supports generality and flexibility. We show that management resources that are expended without guidance from the model can actually harm prospects for persistence of the species [6]. The specific predicted T_e_ for any scenario should not be over-interpreted as parameters are surely not measured exactly nor are they static; nevertheless, the *relative* impacts on the overall metapopulation of changing parameters can be predicted [49], and the relative impacts of scenarios can be ranked even without an exact T_e_ as was exemplified for butterflies [54]. Critically, while the context of each marsh dictates the emergent properties of the entire system, our modeling approach can indeed be synthetic and generalizable to other patchily-distributed endangered species. Our model and results thus have relevance to many species in metapopulations subject to climate stressors including American pikas (*Ochotona princeps*), Cabrera voles (*Microtus cabrerae*), Dupont’s larks (*Chersophilus duponti*), and others [55–59]. A data-driven tool that can synthesize predictions and simulated interventions will be valuable to efficiently and effectively manage the numerous species and communities that depend on conservation intervention if they have any chance to survive.

## Supporting information

S1 File Biomark datalogger specifications. Specifications for RFID technology used during this study. All equipment sources through Biomark (Boise, ID).
(DOCX)

S1 Fig Distribution of inter-marsh distances traveled by voles. (TIF)

S1 Table Response variables for model runs using *Metavole.R*. Abbreviations and descriptions of model outputs.
(DOCX)

S2 Table Scenario data inputs for model. Summary of variables adjusted in model *metavole*.*R* to reproduce anthropogenic impact scenarios on a metapopulation of endangered Amargosa voles. Scenarios were based on ecologically relevant impacts to the entire metapopulation (e.g. drought), or patch specific impacts such as wildfire risks and water flow modifications from private property.
(DOCX)

S3 Table Metapopulation response to simulated management scenarios. Mean values (and standard deviations) of metapopulation response variables across classes of scenarios simulated in *metavole*.*R* to predict impacts of habitat stressors or intervention on extinction risk. Values for F-statistic and *p*-value of ANOVA are included. Letters indicate significant differences between means for each scenario.
(DOCX)
