# Supplementary material for: A stochastic structured metapopulation model to assess recovery scenarios of patchily distributed endangered species: Case study for a Mojave Desert rodent
Source: PLoS One. 2020 Aug 13;15(8):e0237516. doi: 10.1371/journal.pone.0237516 (PMC7425968; doi:10.1371/journal.pone.0237516)
Supplement: S1 File — Specifications for RFID technology used during this study. All equipment sources through Biomark (Boise, ID). (DOCX) [file pone.0237516.s001.docx]

**S1 File**. **Biomark datalogger specifications.** Specifications for RFID technology used during this study. All equipment sources through Biomark (Boise, ID).

PIT tags and Readers

Biomark 601^TM^ Reader (handheld PIT tag reader)

MK25^TM^ Implant Gun

Pre-loaded, sterile Biomark HPT12^TM^ FDX-B PIT tags

Transceiver Units

IS1001^TM^-12V Reader, Application version 2.5.2 (transceiver unit)

Cord^TM^ Antenna System (weather-proof portable enclosure for IS1001, corded antenna, and Jbox junction between IS1001 and corded antenna)

IS1001 Accessory Board (data logger board indicating power and function of reader)

BioTerm^TM^ Cummunication Program

BioStat^TM^ Communication Software

Transceiver settings:

Reader Network Mode: IS1001 Standalone
