# Supplementary figures and images for: A stochastic structured metapopulation model to assess recovery scenarios of patchily distributed endangered species: Case study for a Mojave Desert rodent
Source: PLoS One. 2020 Aug 13;15(8):e0237516. doi: 10.1371/journal.pone.0237516 (PMC7425968; doi:10.1371/journal.pone.0237516)

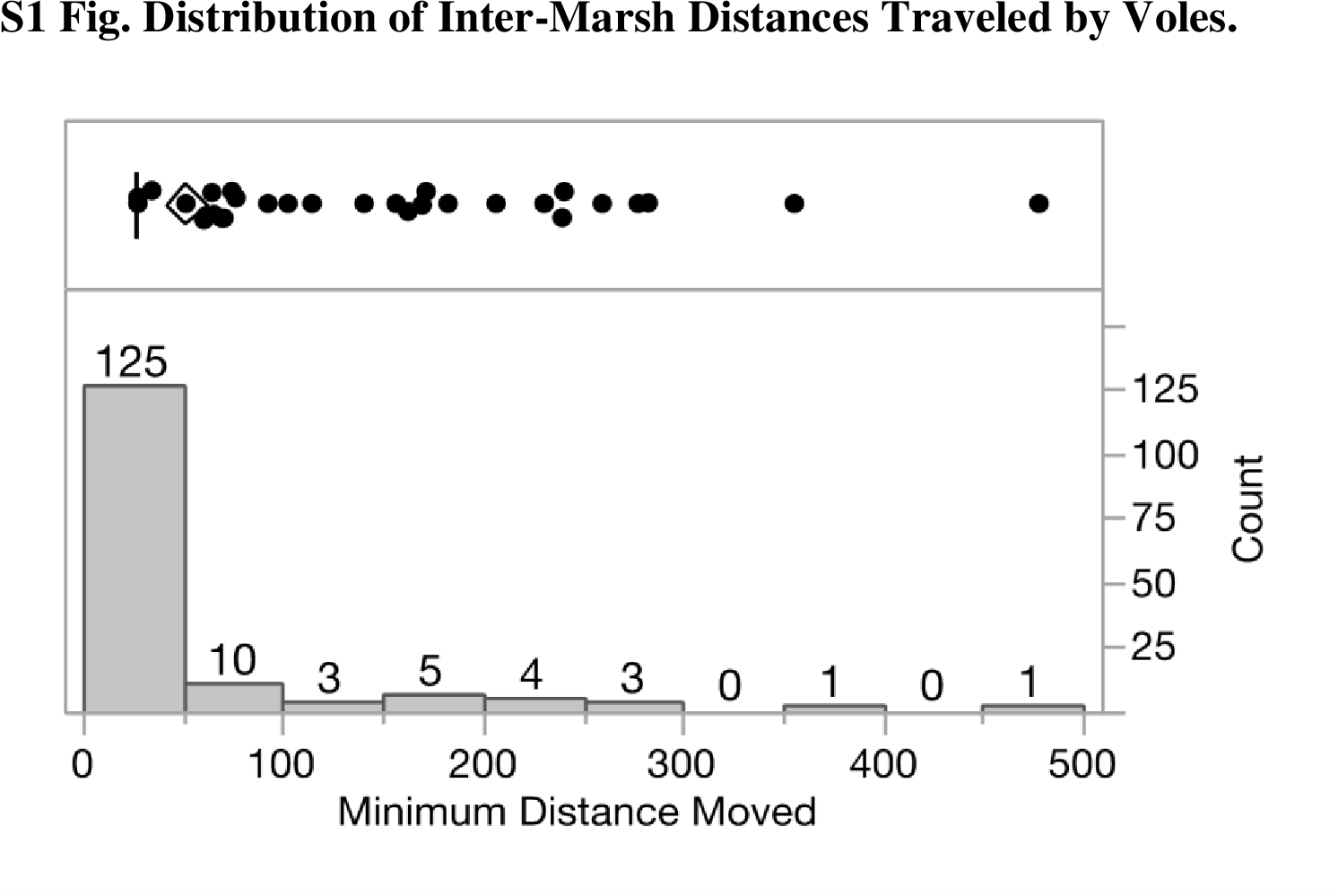

Supplement: S1 Fig — (TIF) [file pone.0237516.s002.tif]
