# Supplementary material for: A stochastic structured metapopulation model to assess recovery scenarios of patchily distributed endangered species: Case study for a Mojave Desert rodent
Source: PLoS One. 2020 Aug 13;15(8):e0237516. doi: 10.1371/journal.pone.0237516 (PMC7425968; doi:10.1371/journal.pone.0237516)
Supplement: S1 Table — Abbreviations and descriptions of model outputs. (DOCX) [file pone.0237516.s003.docx]

**S1 Table. Response Variables for Model Runs Using *Metavole.R*.** Abbreviations and descriptions of model outputs.

| Abbreviation | Meaning |
| --- | --- |
| mvte | Mean time to extinction for the metapopulation |
| mvp25 | Mean proportion of patches occupied at year 25 |
| mvp | Mean proportion of patches occupied at time *t* |
| meanocc | Mean occupancy rate for individual patches in the landscape |
| meanN25 | Mean subpopulation size (per patch) at year 25 |
| meanN | Mean subpopulation size (per patch) at time *t* |
| mN25 | Mean metapopulation size at time 25 |
| mNt | Mean metapopulation size at time *t* |
| mcol25 | Mean number of colonists in the population at time 25 |
| mcolt | Mean number of colonists in the population at time *t* |
