# Supplementary material for: A stochastic structured metapopulation model to assess recovery scenarios of patchily distributed endangered species: Case study for a Mojave Desert rodent
Source: PLoS One. 2020 Aug 13;15(8):e0237516. doi: 10.1371/journal.pone.0237516 (PMC7425968; doi:10.1371/journal.pone.0237516)
Supplement: S2 Table — Summary of variables adjusted in model metavole.R to reproduce anthropogenic impact scenarios on a metapopulation of endangered Amargosa voles. Scenarios were based on ecologically relevant impacts to the entire metapopulation (e.g. drought), or patch specific impacts such as wildfire risks and water flow modifications from private property. (DOCX) [file pone.0237516.s004.docx]

**S2 Table.** **Scenario Data Inputs for Model**. Summary of data input in model *metavole.R* to reproduce anthropogenic impact scenarios on a metapopulation of endangered Amargosa voles. Scenarios were based on ecologically relevant impacts to the entire metapopulation (e.g. drought), or patch specific impacts such as wildfire risks and water flow modifications from private property.

| **Scenario** | **Level** | **Variable adjusted** | **Description** |
| --- | --- | --- | --- |
| Baseline | baseline | none | No changes to landscape or model inputs |
| Drought | avr5 | area, water volume | Minimal drought scenario with 5% reduction in marsh size and water volume landscape-wide |
|  | avr15 | area, water volume | Moderate drought scenario with 15% reduction in marsh size and water volume landscape-wide |
|  | avr35 | area, water volume | Moderate drought scenario with 35% reduction in marsh size and water volume landscape-wide |
|  | avr50 | area, water volume | Severe drought scenario with 50% reduction in marsh size and water volume landscape-wide |
| Fire | firenorth | area, water volume | Marshes 1 and 54 (north) reduced to area of 0.0001 hectare and water volume recalculated |
|  | fire17_21 | area, water volume | Marshes 17 and 21 reduced to area of 0.0001 hectare and water volume recalculated |
|  | firemid | area, water volume | Marshes 13, 66, 55, 52, 34, and 6 (many mid-habitat marshes) reduced to area of 0.0001 hectare and water volume recalculated |
|  | firemid_extr | area, water volume | Marshes 13, 66, 55, 52, 34, 6, 39, 12, 35, and 69 (all mid-habitat marshes) reduced to area of 0.0001 hectare and water volume recalculated |
|  | firesouth_all | area, water volume | Marshes 9, 11, 22, 8, 57, 58, and 36 (south) reduced to area of 0.0001 hectare and water volume recalculated |
| Selective Water Loss | no7_10 | area, water volume | Marshes dependent on Dodge City Spring (Marshes 7 and 10) removed by reducing area to 0.0001 ha and recalculating water volume |
|  | noelias | area, water volume | Marshes dependent on gray water sources from homes (17, 21, and 5) removed by reducing area to 0.0001 ha and recalculating water volume |
|  | nocounty | area, water volume | Marshes dependent on gray water sources from county baths (13, 16, 55, 65 34, and 52) removed by reducing area to 0.0001 ha and recalculating water volume |
|  | nodelights | area, water volume | Marshes dependent on privately owned spring (72, 15, 19, 6, and 66) removed by reducing area to 0.0001 ha and recalculating water volume |
| Megamarsh | normega2 | area, water volume, marsh, northing, easting | Boundaries of northern range marshes 21 and 54 expanded to encompass Marshes 1,7, and 10. Marshes 1, 7, 10 removed from landscape. Several marshes in northern habitat range increased in size. Water volume recalculated based on weighted average of component marshes. New northings and eastings calculated based on adjusted centroid. |
|  | midmega1 | area, water volume, marsh, northing, easting | Large central megamarsh boundary centered within and encompassing Marshes 13, 66, 55, 52, and 34 added to landscape. Water volume recalculated based on weighted average of component marshes. New northings and eastings calculated based on adjusted centroid. |
|  | somega1 | area, water volume, marsh, northing, easting | Marsh 22 expanded to encompass footprint of southern Marshes 9, 11, 27, and 67; Marshes 9, 11, 27, and 67 removed from landscape. Water volume recalculated based on weighted average of component marshes. New northings and eastings calculated based on adjusted centroid. |
|  | somega2 | area, water volume, marsh, northing, easting | Marsh 8 expanded to encompass footprint of southern Marshes 57, 58, and 36; Marshes 57, 58, and 36 removed from landscape. One cluster of marshes in southern habitat range increased in size. Water volume recalculated based on weighted average of component marshes. New northings and eastings calculated based on adjusted centroid. |
| Drought + Fire | dfirenorth | area, water volume | Same as firenorth scenario above, plus all other marshes with area, radius, and water volume reduced by 35%. |
|  | dfire17_21 | area, water volume | Same as fire17_121 scenario above, plus all other marshes with area, and water volume reduced by 35%. |
|  | dfiremid | area, water volume | Same as firemid scenario above, plus all other marshes with area, and water volume reduced by 35%. |
|  | dfiremid_extr | area, water volume | Same as firemid_extr scenario above, plus all other marshes with area, and water volume reduced by 35%. |
|  | dfiresouth_all | area, water volume | Same as firesouth_all scenario above, plus all other marshes with area, and water volume reduced by 35%. |
| Drought + Water Loss | dno7_10 | area, water volume | Same as no7_10 scenario above, plus all other marshes with area, and water volume reduced by 35%. |
|  | dnoelias | area, water volume | Same as noelias scenario above, plus all other marshes with area, and water volume reduced by 35%. |
|  | dnocounty | area, water volume | Same as nocounty scenario above, plus all other marshes with area, and water volume reduced by 35%. |
|  | dnodleights | area, water volume | Same as nodelights scenario above, plus all other marshes with area, and water volume reduced by 35%. |
| Drought + Megamarsh | dnormega2 | area, water volume, marsh, northing, easting | Same as normega2 scenario above, plus all marsh areas, and water volumes reduced by 35%. |
|  | dmidmega1 | area, water volume, marsh, northing, easting | Same as midmega1 scenario above, plus all marsh areas, and water volumes reduced by 35%. |
|  | dsomega1 | area, water volume, marsh, northing, easting | Same as somega1 scenario above, plus all marsh areas, and water volumes reduced by 35%. |
|  | dsomega2 | area, water volume, marsh, northing, easting | Same as somega2 scenario above, plus all marsh areas, and water volumes reduced by 35%. |
