# Supplementary material for: A stochastic structured metapopulation model to assess recovery scenarios of patchily distributed endangered species: Case study for a Mojave Desert rodent
Source: PLoS One. 2020 Aug 13;15(8):e0237516. doi: 10.1371/journal.pone.0237516 (PMC7425968; doi:10.1371/journal.pone.0237516)
Supplement: S3 Table — Mean values (and standard deviations) of metapopulation response variables across classes of scenarios simulated in metavole.R to predict impacts of habitat stressors or intervention on extinction risk. Values for F-statistic and p-value of ANOVA are included. Letters indicate significant differences between means for each scenario. (DOCX) [file pone.0237516.s005.docx]

**S3 Table. Metapopulation Response to Simulated Management Scenarios.** Mean values (and standard deviations) of metapopulation response variables across individual scenarios simulated in *metavole.R* to predict impacts of habitat stressors or intervention on extinction risk. Values for F-statistic and *p*-value of ANOVA are included. Letters indicate significant differences across all scenarios.

|  |  | Mean time to extinction, T_e_ | | Mean fraction patches occupied, t=25 | | Mean total population size, t=25 | | Mean number of colonists, t=25 | |
| --- | --- | --- | --- | --- | --- | --- | --- | --- | --- |
| **Baseline** | | |  |  |  |  |  |  |  |
|  | Status Quo | 66.9^cd^ | ±1.6 | 0.14^b^ | ±0.006 | 82.8^b^ | ±5.5 | 0.43^b^ | ±0.05 |
| **Wildfire** | | |  |  |  |  |  |  |  |
|  | Northern | 60.9^ef^ | ±3.6 | 0.12^ef^ | ±0.006 | 69.9^ef^ | ±3.9 | 0.44^ef^ | ±0.08 |
|  | Marsh 17 & 21 | 63.1^def^ | ±1.6 | 0.12^cedf^ | ±0.004 | 74.6^cdef^ | ±8.2 | 0.37^cdef^ | ±0.04 |
|  | Central | 59.8^ef^ | ±2.9 | 0.10^g^ | ±0.003 | 70.6^g^ | ±6.7 | 0.18^defg^ | ±0.08 |
|  | Central Extreme | 58.4fg | ±1.1 | 0.09^hijk^ | ±0.004 | 65.8^hijk^ | ±6.5 | 0.12^hijk^ | ±0.04 |
|  | Southern | 63.6def | ±3.4 | 0.12^def^ | ±0.005 | 71.9^def^ | ±6.7 | 0.39^def^ | ±0.06 |
| **Selective water loss** | | |  |  |  |  |  |  |  |
|  | No Dodge Spring | 64.1^de^ | ±1.8 | 0.13^bcd^ | ±0.008 | 80.2^bcd^ | ±6.7 | 0.40^bcd^ | ±0.03 |
|  | No Elias Road | 64.7^de^ | ±0.2 | 0.12^cdef^ | ±0.004 | 78.6^cdef^ | ±4.0 | 0.43^cdef^ | ±0.11 |
|  | No County | 59.9^ef^ | ±3.1 | 0.09^gh^ | ±0.003 | 64.6^gh^ | ±2.7 | 0.13^gh^ | ±0.02 |
|  | No Delight’s | 64.6^de^ | ±1.1 | 0.12^cdef^ | ±0.003 | 74.2^cdef^ | ±4.8 | 0.40^cdef^ | ±0.04 |
| **Megamarshes** | | |  |  |  |  |  |  |  |
|  | Northern | 81.8^a^ | ±2.9 | 0.17^a^ | ±0.004 | 342.0^a^ | ±17.0 | 0.66^a^ | ±0.09 |
|  | Central (mid) | 62.6^def^ | ±1.5 | 0.13^bcde^ | ±0.010 | 83.8^bcde^ | ±7.2 | .22^bcde^ | ±0.08 |
|  | Southern 1 | 74.5^b^ | ±1.0 | 0.16^a^ | ±0.004 | 192.9^a^ | ±6.9 | 0.41^a^ | ±0.04 |
|  | Southern 2 | 72.0^bc^ | ±2.6 | 0.17^a^ | ±0.006 | 135.5^a^ | ±10.2 | 0.41^a^ | ±0.09 |
| **Drought** | | |  |  |  |  |  |  |  |
|  | 5% | 62.3 | ±1.1 | 0.13 | ±0.003 | 72.6 | ±5.3 | 0.44 | ±0.09 |
|  | 15% | 60.5 | ±2.9 | 0.11 | ±0.007 | 64.8 | ±6.1 | 0.32 | ±0.10 |
|  | 35% | 54.6^gh^ | ±2.4 | 0.09^ghi^ | ±0.004 | 45.4^ghi^ | ±2.0 | 0.20^ghi^ | ±0.05 |
|  | 50% | 49.6 | ±0.9 | 0.07 | ±0.006 | 29.0 | ±2.5 | 0.13 | ±0.07 |
| **Drought + Wildfire** | | |  |  |  |  |  |  |  |
|  | Northern | 51.8^h^ | ±2.6 | 0.08^kl^ | ±0.005 | 36.5^kl^ | ±2.7 | 0.23^kl^ | ±0.07 |
|  | Marsh 17 & 21 | 52.2^h^ | ±3.4 | 0.08^jk^ | ±0.006 | 39.0^jk^ | ±4.8 | 0.24^jk^ | ±0.07 |
|  | Central | 50.4^h^ | ±2.4 | 0.07^lm^ | ±0.002 | 39.2l^m^ | ±2.1 | 0.10^lm^ | ±0.03 |
|  | Central Extreme | 51.0^h^ | ±1.3 | 0.06^m^ | ±0.004 | 38.0^m^ | ±2.3 | 0.07^m^ | ±0.03 |
|  | Southern | 53.2^h^ | ±2.7 | 0.09^hijk^ | ±0.006 | 42.6^hijk^ | ±5.1 | 0.20^hijk^ | ±0.08 |
| **Drought + Selective water loss** | | |  |  |  |  |  |  |  |
|  | No Dodge Spring | 53.7^gh^ | ±1.6 | 0.09^ghij^ | ±0.004 | 46.0^ghij^ | ±4.0 | 0.23^ghij^ | ±0.04 |
|  | No Elias Road | 52.7^h^ | ±1.3 | 0.08^jk^ | ±0.003 | 41.6^jk^ | ±1.7 | 0.23^jk^ | ±0.05 |
|  | No County | 52.1^h^ | ±2.1 | 0.06^m^ | ±0.003 | 37.4^m^ | ±3.6 | 0.06^m^ | ±0.04 |
|  | No Delight’s | 52.4^h^ | ±2.0 | 0.08^ijk^ | ±0.004 | 40.9^ijk^ | ±2.6 | 0.22^ijk^ | ±0.06 |
| **Drought + Megamarshes** | | |  |  |  |  |  |  |  |
|  | Northern | 76.1^ab^ | ±0.9 | 0.12^cdef^ | ±0.003 | 227.2^cdef^ | ±22.6 | 0.37^cdef^ | ±0.08 |
|  | Central (mid) | 64.3^de^ | ±2.3 | 0.14^bc^ | ±0.006 | 86.3^bc^ | ±4.8 | 0.20^bc^ | ±0.05 |
|  | Southern 1 | 65.5^de^ | ±0.7 | 0.12^cdef^ | ±0.004 | 12.93^cdef^ | ±8.7 | 0.24^cdef^ | ±0.03 |
|  | Southern 2 | 62.61^def^ | ±3.1 | 0.12^f^ | ±0.003 | 77.1^f^ | ±5.8 | 0.23^f^ | ±0.08 |
| ANOVA (*p-*value, F-stat) | | <0.0001, 61.3 | | <0.0001, 189.9 | | <0.0001, 264.4 | | <0.0001, 25.2 | |
